# Live *Bacillus subtilis natto* Promotes Rumen Fermentation by Modulating Rumen Microbiota In Vitro

**DOI:** 10.3390/ani11061519

**Published:** 2021-05-24

**Authors:** Meinan Chang, Fengtao Ma, Jingya Wei, Junhao Liu, Xuemei Nan, Peng Sun

**Affiliations:** State Key Laboratory of Animal Nutrition, Institute of Animal Science, Chinese Academy of Agricultural Sciences, Beijing 100193, China; cmn15567730572@163.com (M.C.); fengtaoma@163.com (F.M.); 18702716486@163.com (J.W.); ljh877460693@163.com (J.L.)

**Keywords:** *Bacillus subtilis natto*, rumen fermentation in vitro, 16S rRNA gene sequencing, volatile fatty acid, rumen microbiota

## Abstract

**Simple Summary:**

Although there is much research on the applications of *Bacillus subtilis natto* in dairy cows, the regulation of it on rumen microorganisms and the mechanisms of microbiota that affect rumen fermentation is still unclear, such as the mechanism of improving ruminal ammonia nitrogen concentration and the pathway of increasing propionic acid production. In this study, we explored the effects of live and autoclaved *B. subtilis natto* on rumen microbiota in vitro by 16S rRNA gene sequencing to clarify the ruminal microbial composition and diversity and their underlying mechanisms.

**Abstract:**

Previous studies have shown that *Bacillus subtilis natto* affects rumen fermentation and rumen microbial community structure, which are limited to detect a few microbial abundances using traditional methods. However, the regulation of *B. subtilis natto* on rumen microorganisms and the mechanisms of microbiota that affect rumen fermentation is still unclear. This study explored the effects of live and autoclaved *B. subtilis natto* on ruminal microbial composition and diversity in vitro using 16S rRNA gene sequencing and the underlying mechanisms. Rumen fluid was collected, allocated to thirty-six bottles, and divided into three treatments: CTR, blank control group without *B. subtilis natto*; LBS, CTR with 10^9^ cfu of live *B. subtilis natto*; and ABS, CTR with 10^9^ cfu of autoclaved *B. subtilis natto*. The rumen fluid was collected after 0, 6, 12, and 24 h of fermentation, and pH, ammonia nitrogen (NH_3_-N), microbial protein (MCP), and volatile fatty acids (VFAs) were determined. The diversity and composition of rumen microbiota were assessed by 16S rRNA gene sequencing. The results revealed LBS affected the concentrations of NH_3_-N, MCP, and VFAs (*p* < 0.05), especially after 12 h, which might be attributed to changes in 18 genera. Whereas ABS only enhanced pH and NH_3_-N concentration compared with the CTR group (*p* < 0.05), which might be associated with changes in six genera. Supplementation with live *B. subtilis natto* improved ruminal NH_3_-N and propionate concentrations, indicating that live bacteria were better than autoclaved ones. This study advances our understanding of *B. subtilis natto* in promoting ruminal fermentation, providing a new perspective for the precise utilization of *B. subtilis natto* in dairy rations.

## 1. Introduction

Probiotics are defined as live microorganisms that are beneficial to the host when administered in adequate amounts [1], and they have been widely used as additives in human food and animal feed [2,3]. Regular ingestion of probiotics as a replacement of chemical feed additives, especially as an alternative to antibiotics, benefits animal health and human food production [4,5]. Among the known probiotics, gram-positive spore-forming bacteria from the genus *Bacillus*, e.g., *Bacillus licheniformis* and *Bacillus subtilis*, have a long history of safe use as probiotic supplements [5,6,7]. Based on the available evidence, *Bacillus* species do not always survive in the anaerobic environment of the gastrointestinal tract because they are aerobic [8,9]. However, because of their spore formation, *Bacillus* species can temporarily survive and proliferate in the digestive tract [10,11,12]. *Bacillus* species inhibit the growth of pathogens such as *Escherichia coli* [13], *Streptococcus* [14], and *Clostridium* [15].

*Bacillus subtilis natto* has been isolated from “natto”, a Japanese fermented soybean staple [16]. Previously, we showed that *B. subtilis natto* should be administered daily to ensure its promotion of rumen fermentation [16,17]. As determined in an in vivo study, the daily administration of *B. subtilis natto* and its culture improves rumen fermentation in dairy cows by promoting the growth of the bacterial rumen biomass and the proteolytic and amylolytic bacteria [17]. Similarly, an in vitro investigation revealed that *B. subtilis natto* (live or autoclaved) increases the abundance of certain bacteria after a 12 h fermentation [16]. These researches only used traditional methods to explore the abundance of a few ruminal microorganisms; nevertheless, 16S rRNA gene sequencing is commonly used to evaluate the relative abundance and diversity of microbiota. Additionally, the regulation of *B. subtilis natto* on rumen microorganisms and the mechanisms of microbiota affecting rumen fermentation is still unclear.

To clarify the relative abundance of ruminal bacteria and their underlying mechanisms, we explored the effects of live and autoclaved *B. subtilis natto* on rumen microbiota in vitro by 16S rRNA gene sequencing. The aim of this study was to delineate the probiotic role of *B. subtilis natto* to direct its future application in the feed industry.

## 2. Materials and Methods

### 2.1. Preparation of Live and Autoclaved B. subtilis natto

*B. subtilis natto* was purchased from the China General Microbiological Culture Collection Center (CGMCC; strain number 1.1086). Live and autoclaved *B. subtilis natto* were prepared as previously described [16]. Briefly, the bacterial inoculum was first incubated in a sterile seed medium at 37 °C for 24 h. Then, an appropriate volume of the culture broth was transferred to a fermentation medium and incubated at 37 °C for 24 h. Colony forming units were determined by spreading culture aliquots on fermentation medium plates; live bacterial cells were centrifuged at 5000× *g* for 10 min at 4 °C using a high-speed freezing centrifuge (Eppendorf 5810R, Eppendorf AG, Hamburg, Germany). Autoclaved bacteria were obtained via steam sterilization at 121 °C for 30 min.

### 2.2. Animals, Diet, and Experimental Design

The Current study was conducted following the principles of the Basel Declaration and Recommendations of the Chinese Academy of Agricultural Sciences Animal Care and Use Committee (Beijing, China). The experimental protocol was approved by the Ethics Committee of the Chinese Academy of Agricultural Sciences (IAS2017-01) (Beijing, China).

Equal volumes of rumen fluid were collected from three healthy multiparous lactating Holstein cows (body weight: 563 ± 9 kg; days in milk: 110 ± 25 d; parity: 2) using a permanent rumen fistula 1 h before morning feeding. The cows were housed in individual tie stalls on the same farm and had free access to water. All the cows were fed the total mixed ration (TMR). The ingredients and chemical composition of the diet are shown in Table 1.

The rumen fluid samples were combined, quickly filtered through four layers of cheesecloth, and then diluted using a buffer solution (1:2, *v*/*v*), prepared as described by Menke and Steingass [18] at 39 °C under a continuous flow of CO_2_. Then they were divided into thirty-six bottles containing 0.5 g TMR as the fermentation substrate; each bottle contained 90 mL of the diluted rumen fluid. They were allocated to three groups: the CTR group, blank control group without *B. subtilis natto*; the LBS group, supplemented 10^9^ cfu live *B. subtilis natto*; the ABS group, supplemented 10^9^ cfu autoclaved *B. subtilis natto*. Live or autoclaved bacteria were added under CO_2_ flow before sealing, and the bottles were incubated at 39 °C with shaking at 150 rpm for 0, 6, 12, and 24 h. The in vitro fermentation was independently conducted three times, and each treatment was performed in triplicate. The fermentation liquid was collected and stored in liquid nitrogen at the indicated times for subsequent determinations of pH, ammonia nitrogen (NH_3_-N), volatile fatty acids (VFAs, including acetate, propionate, butyrate, iso-butyrate, valerate, and iso-valerate), microbial protein (MCP), and rumen microbiota.

### 2.3. Determinations of Ruminal Fermentation Parameters

pH was measured using a portable pH meter (370 model pH meter; Jenway, London, UK). For the other analyses, 25% meta-phosphoric acid was added to the fermentation fluid (1/5, *v*/*v*), and then samples were centrifuged for 10 min at 10,000× *g* at 4 °C using a high-speed freezing centrifuge (Eppendorf 5810R, Eppendorf AG, Hamburg, Germany). The supernatant was collected and stored at −80 °C for NH_3_-N and VFA determinations. Gas chromatography was used to determine VFA levels, as described previously [19]. The NH_3_-N levels were assayed using a modified phenol/hypochlorite method [20]. Rumen MCP levels were determined by using the previously reported purine derivative method [21,22,23]. The MCP levels were calculated from the ratio of purines to N in isolated bacteria. Yeast RNA was used as a standard.

### 2.4. DNA Extraction and 16S rRNA Gene Sequencing of the Rumen Microbiota

Total rumen microbial DNA was extracted using a commercial DNA kit (MP Biomedicals, Santa Ana, CA, USA), strictly following the manufacturer’s standard protocol. The quality of purified DNA was determined by agarose gel electrophoresis. DNA was quantified using a Qubit 3.0 spectrometer (Invitrogen, Carlsbad, CA, USA). The V3 and V4 regions of bacterial 16S rRNA genes were PCR-amplified using the forward primer (5′-CCTACGGGNGGCWGCAG-3′) and the reverse primer (5′-GACTACHVGGGTATCTAATCC-3′). The 30 μL PCR reaction contained 15 μL 2 × Taq master mix, 1 μL (10 μM) of each forward and reverse primer, 10 to 20 ng genomic DNA, and double-distilled H_2_O. The PCR amplification program consisted of one pre-denaturation cycle at 94 °C for 3 min; five cycles at 94 °C for 30 s, 45 °C for 20 s, and 65 °C for 30 s; 20 cycles at 94 °C for 20 s, 55 °C for 20 s, and 72 °C for 30 s; and a final extension at 72 °C for 5 min. First-round PCR products were used as templates for second-round amplicon enrichment PCR. After quantification, all the amplicons were sequenced using an Illumina MiSeq platform to generate 300 bp paired-end reads. DNA library construction and sequencing were performed by Shanghai Personal Biotechnology Co., Ltd. (Shanghai, China). The obtained raw sequences have been submitted to the NCBI Sequence Read Archive, under the accession number SRP188220.

### 2.5. Bioinformatics Analysis

The data was processed using the Quantitative Insights into Microbial Ecology (QIIME v.1.9.0) software package [24]. Raw sequencing reads with exact matches to the barcodes were assigned to respective samples and identified as valid sequences. The low-quality sequences were filtered through the following criteria: sequences that had a length of < 150 bp, sequences that had average Phred scores of < 20, sequences that contained ambiguous bases, and sequences that contained mononucleotide repeats of > 8 bp. FLASH was used to assemble the paired-end reads generated from the DNA fragments [25]. After chimera detection, the operational taxonomy units (OTUs) were identified using UCLUST and defined as sequences clustered with a similarity cutoff of 97% [26]. OTU taxonomy was determined using the RDP classifier retrained on the Greengenes database v. 13_8, with 0.80 confidence threshold. OTUs containing less than 0.001% of total sequences across all samples were discarded. Alpha diversity was determined using various diversity indices (Chao1, abundance-based coverage estimator (ACE), Shannon, and Simpson indices). Beta diversity was calculated using weighted UniFrac distance and visualized principal coordinate analysis (PCoA). Differences between groups were identified using analysis of similarities (ANOSIM).

### 2.6. Statistical Analysis

The microbial data were normalized by lg (X + 1), where X represents the microbiota abundance. The data were then checked for normality using the UNIVARIATE procedure in SAS 9.4 (SAS Institute, Inc., Cary, NC, USA). All data were analyzed using the repeated measurements with compound symmetry variance and covariance structure using the GLMMIX procedure in SAS 9.4. The repeated measures model accounted for the fixed effects of treatment, time, and the interaction of treatment and time. The data are presented as the least square mean and standard error of the mean. Differences among treatments were tested by Tukey’s multiple range test. A *p*-value < 0.05 was accepted as statistically significant, and *p*-values between 0.05 and 0.10 were considered to represent a statistical trend. Canonical correspondence analysis (CCA) of ruminal fermentation parameters and bacterial community composition at the genus level were integrated using Canoco for Windows 4.5. Pearson’s correlation analysis between the fermentation parameters and the rumen microbiota components was determined using SPSS software (IBM SPSS Statistics 20 for windows).

## 3. Results

### 3.1. Rumen Fermentation Parameters

Rumen fermentation parameters were affected by live and autoclaved *Bacillus subtilis natto* except for acetate/propionate ratio (trt, *p* < 0.05) (Table 2). Rumen pH in the ABS group was higher than that in the CTR group at 6 h and 12 h (*p* < 0.05), and it also increased in the LBS group at 24 h (*p* < 0.05). Compared with CTR, NH_3_-N increased significantly in the ABS groups within 24 h (*p* < 0.05), while it was higher before 6 h after adding live *Bacillus subtilis natto* (*p* < 0.05), with no difference after 12 h (*p* > 0.05). The MCP, acetate, propionate, butyrate, iso-valerate, valerate, and total VFA level were higher in the LBS group compared with the CTR group after 12 h (*p* < 0.05). Iso-butyrate also increased in the LBS group at 12 h (*p* < 0.05). Except for the MCP levels and acetate/propionate ratio, all ruminal fermentation parameters were altered with increasing fermentation time (*p* < 0.01).

### 3.2. Ruminal Bacterial Diversity

**Figure 1:**
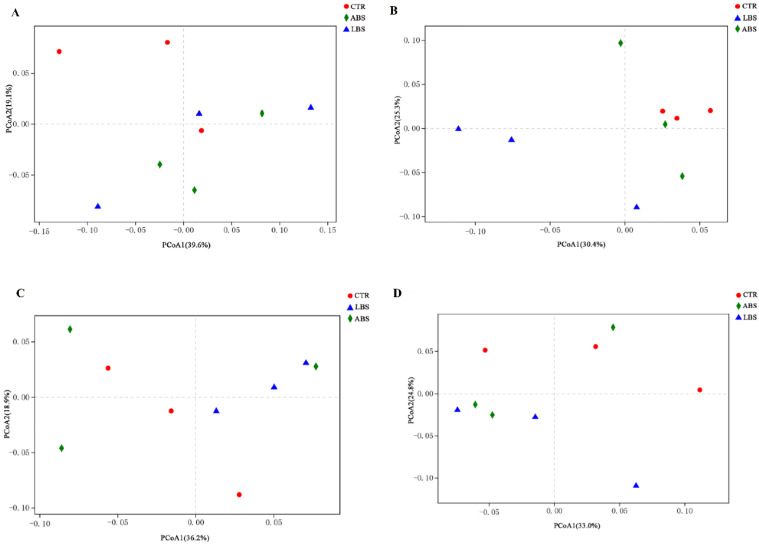
PCoA of the ruminal bacterial community structure in different treatment groups after 0 h (**A**), 6 h (**B**), 12 h (**C**), and 24 h (**D**) fermentation in vitro. The PCoA plot shows microbiota clustering in different groups. Each dot represents an individual sample. Red, blue, and green indicate CTR, LBS, and ABS samples, respectively. CTR, blank control group without *B. subtilis natto*; LBS, CTR with 10^9^ cfu live *B. subtilis natto*; ABS, CTR plus 10^9^ cfu autoclaved *B. subtilis natto*. “Unclassified” refers to sequences that could not be assigned to the genus level.

A total of 2,125,683 sequences were obtained from ruminal fermentation of multiparous lactating Holstein cows in vitro, with an average of 59,046.75 sequences per sample (34,353–87,534 sequences) (Table S1). The Shannon index of the LBS and ABS groups was higher than that of the CTR group at 12 h (*p* < 0.05) (Table 3), and the Simpson index in the LBS group was higher than that in the ABS group (*p* < 0.05). PCoA analysis revealed that the samples from different groups could not be discriminated (*p* > 0.05) (Figure 1 and Table S2), although samples were separated from each other between LBS and CTR groups visually from 6 h to 24 h (Figure 1B–D).

### 3.3. Ruminal Bacterial Community Composition

**Figure 2:**
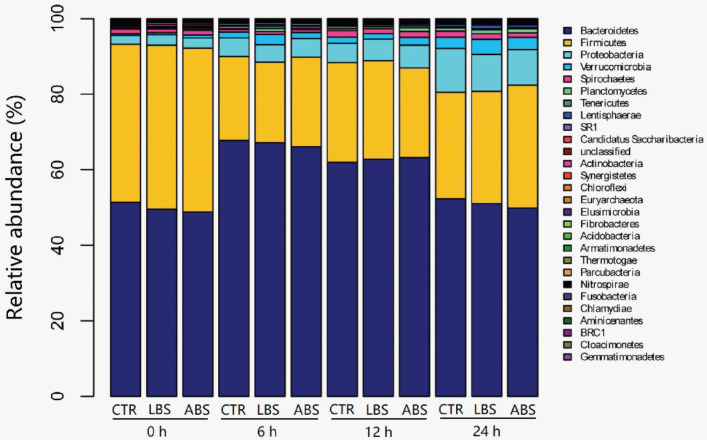
The relative abundances of bacterial phyla. CTR: blank control group without *B. subtilis natto*; LBS: CTR with 10^9^ cfu live *B. subtilis natto*; ABS: CTR with 10^9^ cfu autoclaved *B. subtilis natto*.

Overall, 28 phyla were commonly present in the rumen in vitro fermentation samples from all treatments. Of them, Bacteroidetes was the most dominant phylum in all samples. Firmicutes and Proteobacteria were the 2nd and 3rd dominant phyla (Figure 2). Live *B. subtilis natto* increased the relative abundance of Synergistetes and decreased the relative abundance of Chloroflexi at 24 h, and Elusimicrobia at 12 h compared with the CTR group (*p* < 0.05) (Table 4). The relative abundances of Chloroflexi and Elusimicrobia were reduced in ABS groups at 24 h in comparison with the CTR group (*p* < 0.05).

**Figure 3:**
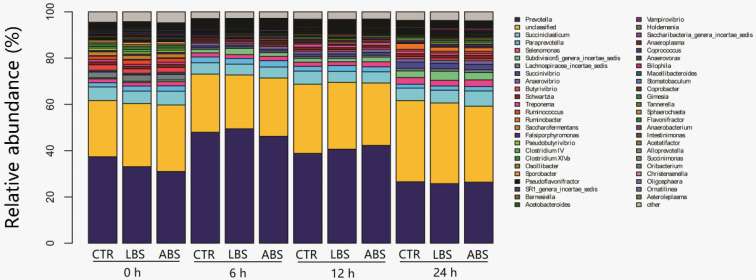
The relative abundance of bacterial genera. CTR: blank control group without *B. subtilis natto*; LBS: CTR with 10^9^ cfu live *B. subtilis natto*; ABS: CTR plus 10^9^ cfu autoclaved *B. subtilis natto*. “Unclassified” refers to sequences that could not be assigned to the genus level.

At the genus level, we identified 463 genera in the three groups and analyzed the top 50 genera, accounting for 95% of the relative abundance of all genera. Of these 50 genera, *Prevotella* was the most dominant genus (Figure 3). The relative abundances of *Prevotella*, *Paraprevotella*, and *Oscillibacter* in the LBS group increased compared with the CTR group before 6 h (*p* < 0.05) (Table 5). In comparison with the CTR treatment, the LBS treatment increased the relative abundances of 11 genera before 12 h, including *Butyrivibrio*, *Ruminococcus*, *Saccharofermentans*, *Pseudobutyrivibrio*, *Clostridium IV*, *Clostridium XIVa*, *Barnesiella*, *Coprococcus*, *Macellibacteroides*, *Succinimonas*, and *Oligosphaera* (*p* < 0.05), and increased the relative abundances of 3 genera within 24 h including *Succinivibrio*, *Bilophila*, and *Sphaerochaeta* (*p* < 0.05). The relative abundance of *Selenomonas* was higher in the LBS group at 6 h and 24 h compared with the other two groups (*p* < 0.05) and was increased numerically at 12 h. Meanwhile, the ABS treatment resulted in an increased relative abundances of *Succinivibrio* and *Succinimonas* within 12 h (*p* < 0.05), *Ruminococcus* at 6 h and 24 h (*p* < 0.05), *Clostridium IV* from 6 h to 24 h (*p* < 0.05), *Bilophila* after 6 h (*p* < 0.05), and *Sphaerochaeta* before 12 h (*p* < 0.05). The relative abundances of *Prevotella*, *Succinivibrio*, *Butyrivibrio*, *Ruminococcus*, *Saccharofermentans*, *Clostridium IV*, *Clostridium XIVa*, *Coprococcus*, *Bilophila*, *Sphaerochaeta*, and *Succinimonas* were affected by time (*p* < 0.05).

### 3.4. Correlation Analysis between Ruminal Bacterial Composition and Fermentation Parameters

**Figure 4:**
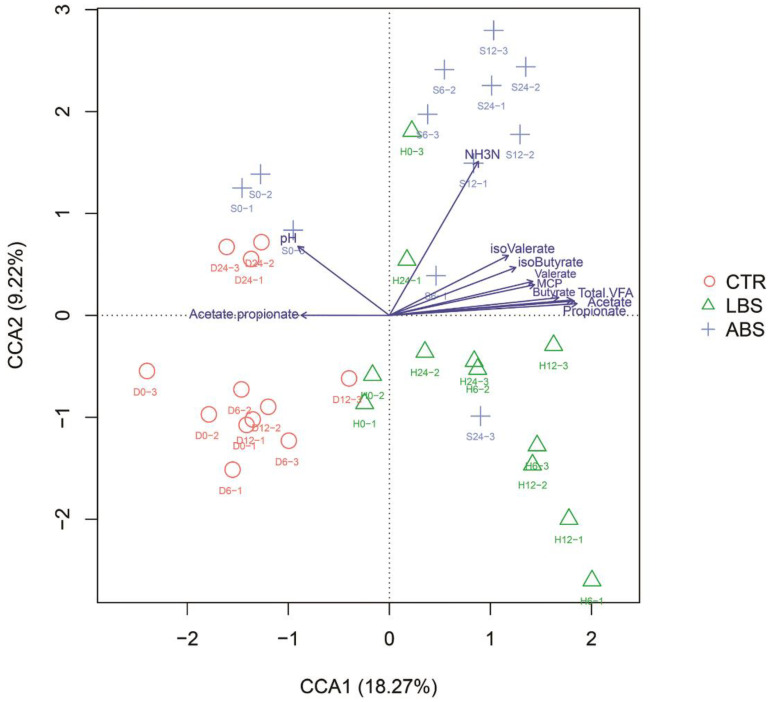
The relationships between ruminal fermentation parameters and the bacterial community composition at the genus level determined by CCA ordination plots. D: CTR: blank control group without *B. subtilis natto*; H: LBS: CTR with 10^9^ cfu live *B. subtilis natto*; S: ABS: CTR with 10^9^ cfu autoclaved *B. subtilis natto*. The symbols “O”, “Δ”, and “+” indicate the bacterial genera in each sample; the arrow indicates ruminal fermentation parameters. The closer the pendulum is to the arrow, the greater the positive correlation between the bacterial genus and fermentation parameters. If the pendulum is positioned relatively far from the arrow, this indicates a negative correlation. If the angle between the arrows is acute, fermentation parameters are positively correlated; the converse indicates a negative correlation.

As shown in the CCA plot (Figure 4), the iso-valerate, iso-butyrate, valerate, butyrate, acetate, propionate, total VFA, and MCP levels were negatively correlated with pH and acetate/propionate ratio. At the same time, they were positively correlated with NH_3_-N and ruminal microorganisms in the LBS group. Microbes in the ABS group were positively correlated with NH_3_-N. The acetate/propionate ratio was positively correlated with microbes in the CTR group.

**Figure 5:**
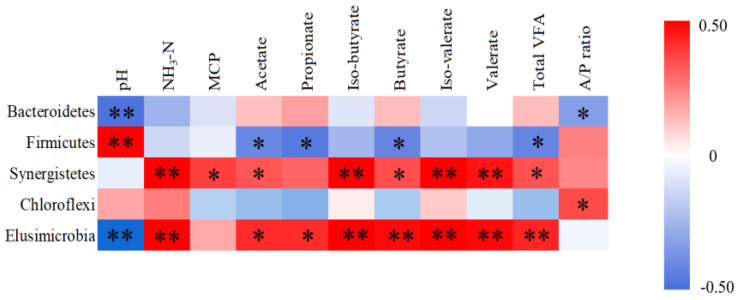
Pearson’s correlation analysis between ruminal fermentation parameters and the three rumen microbiota members at the phylum level. Red and blue titles indicate positive and negative correlations, respectively. A/P ratio: Acetate/propionate ratio. * The correlation is significant at *p* < 0.05. ** The correlation is significant at a level of 0.01.

**Figure 6:**
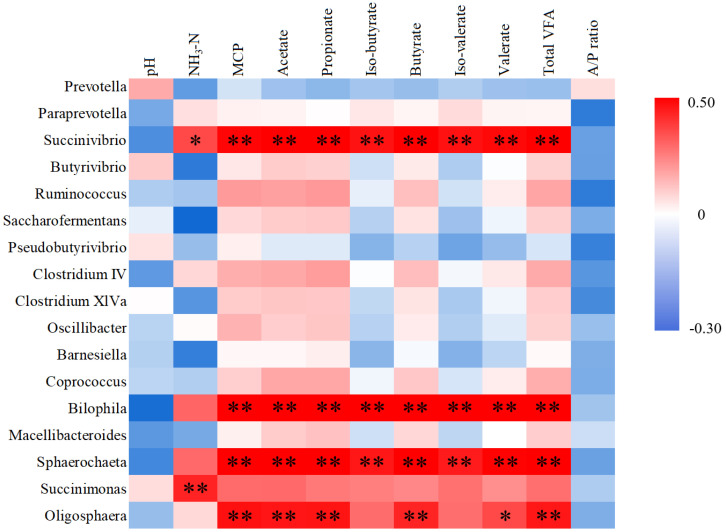
Pearson’s correlation analysis between ruminal fermentation parameters and the 18 differential rumen microbiota members at the genus level. Red and blue titles indicate positive and negative correlations, respectively. A/P ratio: Acetate/propionate ratio. * The correlation is significant at a *p*-value < 0.05. ** The correlation is significant at a level of 0.01.

We also performed an association analysis based on Pearson’s rank correlation coefficient using different taxa. At the phylum level (Figure 5), the phylum Bacteroidetes was negatively correlated with pH and acetate/propionate ratio (*p* < 0.05). The phylum Firmicutes was positively correlated with pH, and negatively correlated with acetate, propionate, butyrate, and total VFA (*p* < 0.05). The phylum Synergistetes was positively correlated with NH_3_-N level, MCP, acetate, iso-butyrate, butyrate, iso-valerate, valerate, and total VFA (*p* < 0.05). The phylum Elusimicrobia was positively correlated with VFAs and NH_3_-N level (*p* < 0.05), and negatively correlated with pH (*p* < 0.05). Chloroflexi was positively correlated with acetate/propionate ratio (*p* < 0.05). At the genus level (Figure 6), *Selenomonas* was positively correlated with VFAs and total VFA (*p* < 0.05). *Succinivibrio* was positively correlated with NH_3_-N level, MCP, VFAs, and total VFA (*p* < 0.05). *Bilophila* and *Sphaerochaeta* were positively correlated with MCP, VFAs, and total VFA (*p* < 0.01). *Oligosphaera* was positively correlated with MCP, acetate, propionate, butyrate, valerate and total VFA (*p* < 0.05). *Succinimonas* was positively correlated with NH_3_-N level (*p* < 0.01).

## 4. Discussion

Ruminal VFA and MCP are produced by microbes [27] and, along with pH and NH_3_-N levels, are important indicators of ruminal function and the stability of the ruminal microecosystem [28,29]. Probiotics can stabilize the ruminal pH [30,31]. Based on in vivo experiments, ingestion of *B. subtilis natto* maintains the rumen pH within a healthy range, fluctuating between 6.20 and 6.50 [7,17]. We observed a similar effect of the CTR and LBS treatments in the current study. The increased pH in the ABS group may be associated with high NH_3_-N levels.

According to early studies, oral administration of *B. subtilis natto* increases growth performance and promotes rumen development in calf [4,32] and early lactation dairy cows [7]. Further, *B. subtilis natto* increases MCP and NH_3_-N levels [16,17], which was confirmed in the current study. MCP is synthesized by microorganisms with NH_3_-N, peptide, and amino acid, which provides rumen bypass protein and allows dairy cows to optimize protein availability [33]. Therefore, the balance between MCP and NH_3_-N is important for dairy cows. *B. subtilis* secretes subtilisin, a proteolytic enzyme [34] that degrades dietary protein in the rumen, providing peptides and amino acids for MCP synthesis. The increased MCP level after 12 h in the LBS group might be attributed to increased NH_3_-N level before 6 h or subtilisin production, which requires further research.

Microbial fermentation and subsequent production of VFAs serve as important sources of energy source to the dairy cow. In addition, VFAs are precursors for the synthesis of milk after absorption by the rumen epithelium. Previous studies have proposed that supplementation of *Bacillus subtilis natto* altered rumen fermentation toward total VFAs, increasing the molar proportion of propionate, iso-butyrate, valerate, and iso-valerate [16,17], which is consistent with the findings of the current study.

The rumen microbial system is a complex natural fermentation system. Ruminants utilize large quantities of fibrous feed via fermentation by rumen microorganisms. Sun et al. [17] reported that the total ruminal bacteria and proteolytic and amylolytic bacteria during *B. subtilis natto* and its culture supplementation were increased, which indicates that *B. subtilis natto* and its culture improve the numbers of rumen bacteria to some extent. Indeed, *B. subtilis* affects the intestinal microbiota of calves and enhances rumen development [35,36]. Furthermore, as determined in vitro, *B. subtilis natto* spores survive in the rumen and can alter rumen fermentation [37]. Similarly, *B. subtilis natto* impacted the ruminal microbiota in the current study. Synergistetes is a minor phylum in the neonatal rumen microbiota besides such major phyla as Bacteroidetes, Firmicutes, and Proteobacteria [38]. It produces peptides and free amino acids by proteolytic degradation [39,40]. Free amino acids are further degraded to produce organic acids and ammonia, promoting the synthesis of NH_3_-N and MCP. Consistent with these findings, in the present study, the relative abundance of Synergistetes increased in the LBS group at 24 h and was also positively correlated with the NH_3_-N and MCP levels. *Elusimicrobium minutum* belongs to the phylum Elusimicrobia, and ferments d-galactose, d-glucose, d-fructose, d-glucosamine, and *N*-acetyl-d-glucosamine, with acetate, ethanol, hydrogen, and alanine as the major products [41], which is consistent with the correlation analysis that Elusimicrobia were positively associated with acetate and total VFA levels. However, they were decreased in the LBS group at 12 h and the ABS group at 24 h, which might retard the production of acetate and result in differences in the acetate/propionate ratio among the three groups.

*Prevotella* is one of the most numerous microbes to be cultured from the rumen and hind-gut of goat and cattle [42]. It participates in the degradation of protein, fiber, hemicellulose, and pectin [43,44]. Live *B. subtilis* secretes cellulase, protease, amylase, and other enzymes, which improve the activity of enzymes in the animal digestive tract and enhance feed efficiency [45,46]. In the current study, the relative abundance of *Prevotella* increased in the LBS treatment before 6 h. Available evidence shows that the relative abundances of *Ruminococcus albus* and *Ruminococcus flavefaciens* increase in weaning calf administered *B. subtilis natto* [47], also promoting the growth of *Butyrivibrio fibrisolvens* [48]. These findings are consistent with the observations of the current study. Namely, the relative abundance of *Ruminococcus* was enhanced in the LBS and ABS groups, with a similar trend for *Butyrivibrio* in the LBS group. *Butyrivibrio*, together with *Pseudobutyrivibrio*, *Oscillibacter*, *Coprococcus*, and *Macellibacteroides*, ferment glucose and polysaccharides to produce butyrate [49,50,51,52]. Further, *Macellibacteroides* ferments cellobiose, glucose, lactose, and other sugars, using them as electron donors to produce iso-butyrate [53]. Increased butyrate and iso-butyrate levels in the LBS group before 12 h might be associated with the growth of these bacteria.

*Clostridium* produces up to 20 times more ammonia than other ammonia-producing ruminal bacteria [54] and may have promoted the synthesis of NH_3_-N in the LBS and ABS groups. As shown in many studies, *Bilophila*, *Sphaerochaeta*, *and Oligosphaera* produce acetic acid via fermenting starch, cellulose, glucose, and other carbohydrates [55,56,57]. We showed that acetate levels had a positive correlation with these bacteria. Furthermore, *Paraprevotella*, *Succinivibrio*, *Saccharofermentans*, *Barnesiella*, and *Succinimonas* use starch and various sugars to produce succinic acid [58,59,60,61,62], which can be decarboxylated by *Selenomonas ruminantium* to produce propionate, a major ruminal VFA [59]. These observations support the finding of the current study that propionate and the relative abundance of *Selenomonas* were higher in the LBS group than that in the CTR group.

**Figure 7:**
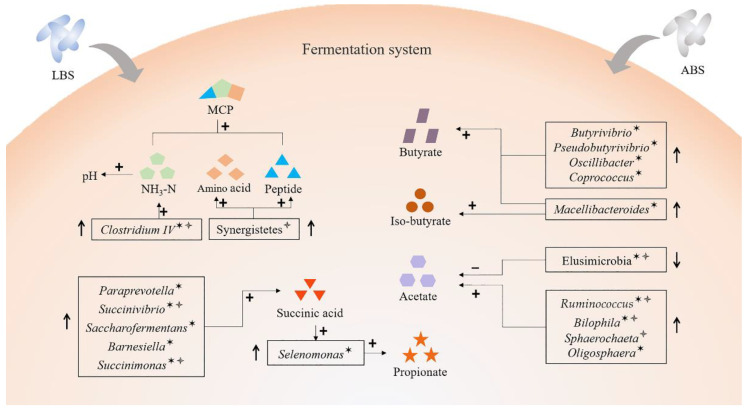
Proposed model of the mechanisms of microbiota affects rumen fermentation after supplemented live and autoclaved *B. subtilis natto*. LBS: live *B. subtilis natto*; ABS: autoclaved *B. subtilis natto*. ^✶^ indicates effects in the LBS group, ^🟇^ indicate effects in the ABS group.

In a nutshell, the in vitro study expounded that the detailed process of *B. subtilis natto* improved rumen fermentation; more precisely, it illustrated the relationship of various differential phyla and genera and ruminal fermentation parameters after supplemented LBS and ABS (Figure 7). For example, the synthesis of ammonia nitrogen might be associated with the genus *Clostridium IV*. The production of the increased propionic acid was mediated by succinic acid, which was produced by five genera and utilized by *Selenomonas*.

## 5. Conclusions

This study demonstrated that live *B. subtilis natto* affected the fermentation parameters except for pH and acetate/propionate, especially after 12 h, which might be attributed to the changes of 18 genera. While ABS only enhanced pH and NH_3_-N concentration compared with the CTR group, which might be associated with the changes of six genera. Supplementation with live *B. subtilis natto* improved ruminal NH_3_-N and propionate concentrations, indicating that live bacteria were better than autoclaved ones. Therefore, these findings advance our understanding of *B. subtilis natto* in promoting ruminal fermentation, providing a new perspective for the precise utilization of *Bacillus subtilis natto* in dairy rations.

## Figures and Tables

**Table 1:** The ingredients and chemical composition of the diet (%, as-fed DM).

Item	%
Ingredient	
Alfalfa hay	16.65
Corn silage	20.25
Soybean meal	8.4
Rapeseed meal	1.3
Cottonseed meal	1.1
Extruded soybean	2.1
Sugarbeet	4.2
Apple pomace	2.1
Whole cottonseed	10.5
DDGS ^1^	2.6
Flaked corn	10.5
Corn	17.1
Fat powder	1.1
Limestone	0.4
Salt	0.4
Premix ^2^	0.5
NaHCO_3_	0.8
Chemical analysis	
CP	15.66
EE	3.45
NDF	26.53
ADF	22.04
Ash	6.11
NE_L_^3^, MJ/kg	6.46

^1^ DDGS: distiller’s dried grains with solubles. ^2^ Premix provided per kg of DM: vitamin A: 770,000 IU; vitamin D_3_: 192,500 IU; vitamin E: 7000 IU; niacin: 700 mg; Cu: 2750 mg; Mn: 4200 mg; Zn: 10,890 mg; I: 110 mg; Se: 132 mg; Co: 88 mg. ^3^ Calculated value (based on China NY/t 34, 2004).

**Table 2:** The effects of live and autoclaved *B. subtilis natto* on pH, NH_3_-N, MCP, and VFAs during ruminal fermentation in vitro.

Item	Treatment ^1^			SEM	*p*-Value		
	CTR	LBS	ABS		Trt ^2^	Time	Trt × Time
**pH**							
0 h	6.62	6.62	6.67	0.02	0.01	<0.01	0.65
6 h	6.46 ^b^	6.48 ^ab^	6.52 ^a^				
12 h	6.49	6.47	6.51				
24 h	6.43 ^b^	6.49 ^a^	6.50 ^a^				
NH_3_-N, mg/dL							
0 h	9.28 ^b^	18.54 ^a^	23.14 ^a^	2.23	<0.01	<0.01	0.31
6 h	16.69 ^b^	22.58 ^a^	25.48 ^a^				
12 h	23.51 ^a^	22.76 ^b^	31.53 ^a^				
24 h	39.21 ^b^	42.28 ^b^	52.23 ^a^				
MCP, mg/mL							
0 h	0.43	0.39	0.46	0.05	0.02	0.11	0.32
6 h	0.38	0.49	0.44				
12 h	0.38 ^b^	0.56 ^a^	0.46 ^ab^				
24 h	0.41 ^b^	0.58 ^a^	0.57 ^a^				
Acetate, mmol/L							
0 h	18.46	25.38	17.45	3.47	<0.01	<0.01	0.14
6 h	31.57	32.72	31.80				
12 h	23.87 ^b^	42.95 ^a^	32.56 ^b^				
24 h	30.27 ^b^	49.60 ^a^	40.08 ^ab^				
Propionate, mmol/L							
0 h	5.92	7.24	5.23	1.01	<0.01	<0.01	0.07
6 h	9.95	10.04	9.88				
12 h	7.02 ^b^	13.34 ^a^	10.28 ^ab^				
24 h	9.42 ^b^	14.46 ^a^	11.70 ^ab^				
Iso-butyrate, mmol/L							
0 h	0.22	0.22	0.17	0.04	0.04	<0.01	0.12
6 h	0.29	0.27	0.28				
12 h	0.21 ^a^	0.41 ^a^	0.34 ^ab^				
24 h	0.40	0.53	0.45				
Butyrate, mmol/L							
0 h	3.15	3.63	2.62	0.56	<0.01	<0.01	0.06
6 h	5.16	5.00	4.89				
12 h	3.73 ^b^	7.09 ^a^	5.50 ^ab^				
24 h	5.36 ^b^	7.97 ^a^	6.32 ^ab^				
Iso-valerate, mmol/L							
0 h	0.33	0.36	0.27	0.07	0.02	<0.01	0.14
6 h	0.46	0.43	0.43				
12 h	0.35 ^b^	0.66 ^a^	0.56 ^ab^				
24 h	0.68 ^b^	0.95 ^a^	0.79 ^ab^				
Valerate, mmol/L							
0 h	0.47	0.47	0.35	0.11	0.02	<0.01	0.04
6 h	0.64	0.58	0.58				
12 h	0.45 ^b^	0.88 ^a^	0.70 ^ab^				
24 h	0.75 ^b^	1.02 ^a^	0.87 ^ab^				
Total VFA, mmol/L							
0 h	28.55	37.32	26.08	5.16	<0.01	<0.01	0.10
6 h	48.08	49.03	47.86				
12 h	35.64 ^b^	65.33 ^a^	49.94 ^ab^				
24 h	46.88 ^b^	74.54 ^a^	60.20 ^ab^				
Acetate/propionate							
0 h	3.13	3.50	3.33	0.08	0.10	0.17	0.09
6 h	3.17	3.26	3.20				
12 h	3.36	3.21	3.18				
24 h	3.21	3.43	3.43				

^a,b^ The letters in the same row with different superscripts are significantly different between treatments (*p* < 0.05). ^1^ Treatments: CTR: blank control group without *B. subtilis natto*; LBS: CTR with 10^9^ cfu live *B. subtilis natto*; ABS: CTR with 10^9^ cfu autoclaved *B. subtilis natto*. ^2^ Abbreviations for each treatment.

**Table 3:** Alpha diversity index (including Chao1, ACE, Shannon, and Simpson indices) for the ruminal microbiota in the three treatment groups.

Item	Treatment ^1^			SEM	*p*-Value		
	CTR	LBS	ABS		Trt ^2^	Time	Trt × Time
**Chao1**							
0 h	4534.84	4369.79	4866.76	273.02	0.47	0.07	0.95
6 h	4105.18	4026.20	4274.72				
12 h	4565.83	4441.05	4717.21				
24 h	4883.78	4687.70	4622.04				
ACE							
0 h	5259.44	4580.10	5257.77	390.63	0.57	0.07	0.53
6 h	4285.27	4663.50	5022.65				
12 h	5255.84	4902.38	5196.32				
24 h	5966.50	5537.83	5158.71				
Shannon							
0 h	5.98	6.13	6.15	0.06	0.05	<0.01	0.29
6 h	5.72	5.73	5.66				
12 h	5.61 ^b^	5.84 ^a^	5.80 ^a^				
24 h	5.73	5.76	5.77				
Simpson							
0 h	0.012	0.009	0.009	0.001	0.03	<0.01	0.35
6 h	0.016	0.014	0.015				
12 h	0.019 ^a^	0.015 ^ab^	0.012 ^b^				
24 h	0.013	0.012	0.013				

^a,b^ The letters in the same row with different superscripts are significantly different between treatments (*p* < 0.05). ^1^ Treatments: CTR: blank control group without *B. subtilis natto*; LBS: CTR with 10^9^ cfu live *B. subtilis natto*; ABS: CTR with 10^9^ cfu autoclaved *B. subtilis natto*. ^2^ Abbreviation for each treatment.

**Table 4:** Profiles of the ruminal bacterial communities among three groups at the rank of phylum according to taxon-based analysis.

Phylum	Treatment ^1^			SEM	*p*-Value		
	CTR	LBS	ABS		Trt ^2^	Time	Trt × Time
Bacteroidetes							
0 h	1.72	1.70	1.69	0.014	0.62	<0.01	0.94
6 h	1.84	1.83	1.83				
12 h	1.80	1.80	1.81				
24 h	1.72	1.72	1.71				
Firmicutes							
0 h	1.63	1.65	1.65	0.024	0.66	<0.01	0.35
6 h	1.36	1.35	1.39				
12 h	1.44	1.43	1.39				
24 h	1.47	1.48	1.53				
Synergistetes							
0 h	0.063	0.068	0.062	0.007	0.18	<0.01	0.06
6 h	0.036	0.033	0.049				
12 h	0.059	0.069	0.049				
24 h	0.085 ^b^	0.100 ^ab^	0.120 ^a^				
Chloroflexi							
0 h	0.053	0.058	0.067	0.006	0.04	<0.01	0.04
6 h	0.036	0.032	0.031				
12 h	0.044	0.025	0.036				
24 h	0.083 ^a^	0.056 ^b^	0.054 ^b^				
Elusimicrobia							
0 h	0.009	0.009	0.014	0.003	0.02	<0.01	0.08
6 h	0.024	0.020	0.018				
12 h	0.041 ^a^	0.028 ^b^	0.032 ^ab^				
24 h	0.044 ^a^	0.037 ^ab^	0.031 ^b^				

^a,b^ The letters in the same row with different superscripts are significantly different between treatments (*p* < 0.05). ^1^ Treatments: CTR: blank control group without *B. subtilis natto*; LBS: CTR with 10^9^ cfu live *B. subtilis natto*; ABS: CTR with 10^9^ cfu autoclaved *B. subtilis natto*. ^2^ Abbreviation for each treatment.

**Table 5:** Differential ruminal bacterial communities among three groups at the rank of genus according to taxon-based analysis.

Genus	Treatment ^1^			SEM	*p*-Value		
	CTR	LBS	ABS		Trt ^2^	Time	Trt × Time
*Prevotella*							
0 h	1.58 ^b^	1.70 ^a^	1.64 ^ab^	0.02	<0.01	<0.01	<0.01
6 h	1.53 ^b^	1.67 ^a^	1.44 ^c^				
12 h	1.50 ^ab^	1.60 ^a^	1.43 ^b^				
24 h	1.69 ^a^	1.62 ^a^	1.44 ^b^				
*Paraprevotella*							
0 h	0.45 ^b^	0.54 ^a^	0.50 ^ab^	0.02	<0.01	0.19	<0.01
6 h	0.50 ^b^	0.57 ^a^	0.42 ^c^				
12 h	0.52 ^a^	0.49 ^ab^	0.46 ^b^				
24 h	0.55 ^a^	0.54 ^a^	0.46 ^ab^				
*Selenomonas*							
0 h	0.44	0.50	0.42	0.03	<0.01	0.50	0.53
6 h	0.40 ^b^	0.59 ^a^	0.45 ^b^				
12 h	0.44	0.54	0.45				
24 h	0.44 ^b^	0.59 ^a^	0.44 ^b^				
*Succinivibrio*							
0 h	0.06 ^c^	0.35 ^a^	0.23 ^b^	0.03	<0.01	<0.01	<0.01
6 h	0.06 ^c^	0.58 ^a^	0.26 ^b^				
12 h	0.09 ^c^	0.54 ^a^	0.32 ^b^				
24 h	0.27 ^b^	0.51 ^a^	0.35 ^b^				
*Butyrivibrio*							
0 h	0.24 ^b^	0.53 ^a^	0.25 ^b^	0.03	<0.01	<0.01	<0.01
6 h	0.22 ^b^	0.44 ^a^	0.29 ^b^				
12 h	0.22 ^b^	0.49 ^a^	0.29 ^b^				
24 h	0.24 ^ab^	0.18 ^b^	0.29 ^a^				
*Ruminococcus*							
0 h	0.15 ^b^	0.40 ^a^	0.15 ^b^	0.02	<0.01	<0.01	<0.01
6 h	0.18 ^b^	0.47 ^a^	0.25 ^a^				
12 h	0.18 ^b^	0.42 ^a^	0.24 ^b^				
24 h	0.16 ^b^	0.17 ^b^	0.27 ^a^				
*Saccharofermentans*							
0 h	0.18 ^b^	0.43 ^a^	0.15 ^b^	0.02	<0.01	<0.01	<0.01
6 h	0.20 ^b^	0.44 ^a^	0.13 ^c^				
12 h	0.17 ^b^	0.43 ^a^	0.15 ^b^				
24 h	0.19	0.16	0.16				
*Pseudobutyrivibrio*							
0 h	0.16 ^b^	0.30 ^a^	0.20 ^b^	0.02	<0.01	0.06	<0.01
6 h	0.17 ^b^	0.30 ^a^	0.24 ^a^				
12 h	0.18 ^b^	0.28 ^a^	0.23 ^ab^				
24 h	0.21 ^a^	0.13 ^b^	0.22 ^a^				
*Clostridium IV*							
0 h	0.12 ^b^	0.22 ^a^	0.15 ^b^	0.02	<0.01	<0.01	<0.01
6 h	0.15 ^b^	0.29 ^a^	0.24 ^a^				
12 h	0.19 ^b^	0.27 ^a^	0.23 ^a^				
24 h	0.15 ^b^	0.14 ^b^	0.25 ^a^				
*Clostridium XIVa*							
0 h	0.12 ^b^	0.33 ^a^	0.15 ^b^	0.01	<0.01	<0.01	<0.01
6 h	0.15 ^b^	0.34 ^a^	0.15 ^b^				
12 h	0.13 ^b^	0.32 ^a^	0.17 ^b^				
24 h	0.14 ^b^	0.12 ^b^	0.18 ^a^				
*Oscillibacter*							
0 h	0.13 ^b^	0.25 ^a^	0.14 ^b^	0.03	<0.01	0.32	<0.01
6 h	0.16 ^b^	0.28 ^a^	0.17 ^b^				
12 h	0.16	0.23	0.18				
24 h	0.16	0.13	0.22				
*Barnesiella*							
0 h	0.10 ^b^	0.20 ^a^	0.10 ^b^	0.02	<0.01	0.21	0.02
6 h	0.13 ^b^	0.22 ^a^	0.07 ^b^				
12 h	0.11 ^b^	0.19 ^a^	0.09 ^b^				
24 h	0.13	0.10	0.09				
*Coprococcus*							
0 h	0.069 ^b^	0.17 ^a^	0.08 ^b^	0.009	<0.01	<0.01	<0.01
6 h	0.069 ^c^	0.19 ^a^	0.10 ^b^				
12 h	0.093 ^b^	0.18 ^a^	0.10 ^b^				
24 h	0.095 ^a^	0.06 ^b^	0.12 ^a^				
*Bilophila*							
0 h	0.05 ^b^	0.10 ^a^	0.04 ^b^	0.012	<0.01	<0.01	<0.01
6 h	0.06 ^b^	0.17 ^a^	0.05 ^b^				
12 h	0.05 ^c^	0.21 ^a^	0.10 ^b^				
24 h	0.04 ^c^	0.21 ^a^	0.13 ^b^				
*Macellibacteroides*							
0 h	0.07 ^b^	0.15 ^a^	0.07 ^b^	0.02	<0.01	0.35	0.02
6 h	0.10 ^b^	0.19 ^a^	0.05 ^b^				
12 h	0.09 ^b^	0.17 ^a^	0.08 ^b^				
24 h	0.12	0.08	0.07				
*Sphaerochaeta*							
0 h	0.02 ^c^	0.09 ^a^	0.05 ^b^	0.006	<0.01	<0.01	<0.01
6 h	0.03 ^c^	0.12 ^a^	0.06 ^b^				
12 h	0.04 ^c^	0.14 ^a^	0.09 ^b^				
24 h	0.06 ^b^	0.11 ^a^	0.07 ^b^				
*Succinimonas*							
0 h	0.014 ^b^	0.068 ^a^	0.077 ^a^	0.008	<0.01	0.03	0.08
6 h	0.023 ^b^	0.077 ^a^	0.069 ^a^				
12 h	0.021 ^b^	0.064 ^a^	0.065 ^a^				
24 h	0.061	0.082	0.068				
*Oligosphaera*							
0 h	0.0086 ^b^	0.064 ^a^	0.033 ^ab^	0.016	<0.01	0.28	0.65
6 h	0.0086 ^b^	0.093 ^a^	0.022 ^ab^				
12 h	0.017 ^b^	0.120 ^a^	0.045 ^b^				
24 h	0.027	0.079	0.032				

^a,b,c^ The letters in the same row with different superscripts are significantly different between treatments (*p* < 0.05). ^1^ Treatments: CTR: blank control group without *B. subtilis natto*; LBS: CTR with 10^9^ cfu live *B. subtilis natto*; ABS: CTR with 10^9^ cfu autoclaved *B. subtilis natto*. ^2^ Abbreviations for each treatment.

## Data Availability

The 16S rRNA data of rumen fluid samples are available at the National Center for Biotechnology Information (NCBI) Sequence Read Archive under the accession number SRP188220.

## Supplementary Materials

The following are available online at https://www.mdpi.com/article/10.3390/ani11061519/s1, Table S1: Number of sequences in samples at different time points from each treatment, Table S2: Number of sequences in samples at different time points from each treatment.
