# Supplementary material for: Live Bacillus subtilis natto Promotes Rumen Fermentation by Modulating Rumen Microbiota In Vitro
Source: Animals (Basel). 2021 May 24;11(6):1519. doi: 10.3390/ani11061519 (PMC8225115; doi:10.3390/ani11061519)
Supplement: Supplementary file 1 [file animals-11-01519-s001.zip › animals-1193960-supplementary.pdf]

Supplementary Materials

Table S1. Number of sequences in samples at different time points from each treatment.

**Treatment[1]**	**Time (h)**	**Sequence number (bp)**
CTR	0	53,778
CTR	0	46,174
CTR	0	56,306
CTR	6	52,571
CTR	6	61,593
CTR	6	62,974
CTR	12	47,295
CTR	12	52,339
CTR	12	56,431
CTR	24	53,672
CTR	24	85,494
CTR	24	61,526
LBS	0	62,583
LBS	0	40,620
LBS	0	56,168
LBS	6	54,143
LBS	6	71,162
LBS	6	60,743
LBS	12	82,373
LBS	12	41,337
LBS	12	**34,353**
LBS	24	47,853
LBS	24	64,572
LBS	24	73,891
ABS	0	61,930
ABS	0	61,322
ABS	0	59,173
ABS	6	63,209
ABS	6	66,612
ABS	6	56,023
ABS	12	45,121
ABS	12	60,030
ABS	12	59,633
ABS	24	67,098
ABS	24	**87,534**
ABS	24	58,047

Mean sequence (bp)	59,046.75
Total sequence(bp)	2,125,683

[1]Treatments consisted of blank control (CTR) with no additive, LBS with 10^9 cfu live *Bacillus subtilis natto*, ABS with a minimum of 10^9 cfu autoclaved *Bacillus subtilis natto*.

Table S2. Number of sequences in samples at different time points from each treatment.

ANOSIM analysis	CTR vs. LBS	CTR vs. ABS
**0 h**		
R	-0.11	-0.11
P-value	0.69	0.69
**6 h**		
R	-0.15	-0.11
P-value	0.91	0.59
**12 h**		
R	-0.11	0.52
P-value	0.83	0.10
**24 h**		
R	-0.04	0.00
P-value	0.60	0.50
